# Performance evaluation of computerized antepartum fetal heart rate monitoring: Dawes–Redman algorithm at term

**DOI:** 10.1002/uog.29167

**Published:** 2025-02-02

**Authors:** G. Davis Jones, B. Albert, W. Cooke, M. Vatish

**Affiliations:** ^1^ Oxford Digital Health Labs, Nuffield Department of Women's & Reproductive Health University of Oxford, Women's Centre, John Radcliffe Hospital Oxford UK; ^2^ The Alan Turing Institute London UK

**Keywords:** algorithm evaluation, antepartum, cardiotocography, clinical decision‐making, electronic fetal monitoring, high‐risk pregnancy, non‐stress test, obstetrics, predictive value of tests, prevalence effect

## Abstract

**Objectives:**

To assess the effectiveness of the Dawes–Redman algorithm in identifying fetal wellbeing at term by analyzing 30 years of retrospective clinical data, comparing normal and adverse pregnancy outcomes, evaluating key metrics and testing its performance when used 0–48 h before delivery.

**Methods:**

Antepartum fetal heart rate (FHR) traces from term singleton pregnancies at 37 + 0 to 41 + 6 weeks' gestation obtained between 1991 and 2024 were extracted from the Oxford University Hospitals database. Traces with > 30% of their signal information missing or with incomplete Dawes–Redman analyses were excluded. Only traces performed within 48 h prior to delivery were considered. A cohort of pregnancies with subsequent normal pregnancy outcome (NPO) was established using rigorous inclusion and exclusion criteria. Another cohort of pregnancies with adverse pregnancy outcome (APO) was developed if the neonate experienced at least one of seven APOs after delivery. Propensity score matching (PSM) facilitated a balanced comparison between NPO and APO cohorts using six factors: gestational age at FHR monitoring, fetal sex, maternal body mass index at presentation, maternal age at delivery, parity and time interval between FHR trace and delivery. FHR traces were categorized as either ‘criteria met’ (indicating fetal wellbeing) or ‘criteria not met’ (indicating a need for further evaluation) according to the Dawes–Redman algorithm, which informed the evaluation of predictive performance metrics. Performance was assessed using accuracy, sensitivity, specificity, positive predictive value, and negative predictive value (NPV) adjusted for various population risk prevalences of APO.

**Results:**

A balanced dataset of 3316 antepartum FHR traces was developed with PSM (standardized mean difference < 0.10). The Dawes–Redman algorithm showed a high specificity of 90.7% (95% CI, 89.2–92.0%) for ruling out APO. Sensitivity was 18.2% (95% CI, 16.3–20.0%). The NPV varied with the population prevalence of APO and was high in very‐low‐risk settings (NPV, 99.1% (95% CI, 98.9–99.3%) at 1% APO prevalence) and decreased with increasing risk of APO (NPV, 72.1% (95% CI, 67.7–76.1%) at 30% APO prevalence). Temporal proximity of FHR assessment to delivery indicated robust specificity, which was similar for assessments performed at 0–24 h and 24–48 h prior to delivery (specificity at 0–24 h, 90.8% (95% CI, 88.8–92.7%); specificity at 24–48 h, 90.3% (95% CI, 88.2–92.3%); *P* = 0.898). Across the different adverse outcomes comprising the APO cohort, the performance of the Dawes–Redman algorithm remained consistent, with high specificity (ranging from 87.7% to 94.7%) and NPVs (ranging from 95.4% to 96.0%), confirming its utility in identifying fetal wellbeing.

**Conclusion:**

These findings indicate that the Dawes–Redman algorithm is effective for its intended purpose: identifying a state of fetal wellbeing. This is evidenced by its high specificity. However, its low sensitivity suggests limitations in its ability to identify fetuses at risk of APO. The predictive accuracy of the algorithm is affected significantly by the prevalence of healthy pregnancies within the population. Clinical interpretation of FHR traces that do not satisfy the 10 Dawes–Redman criteria warrant further expert clinical evaluation. While the algorithm proves reliable for its primary objective, the development of an algorithm optimized for high‐risk pregnancy scenarios remains an area of interest for future study. © 2025 The Author(s). *Ultrasound in Obstetrics & Gynecology* published by John Wiley & Sons Ltd on behalf of International Society of Ultrasound in Obstetrics and Gynecology.

## INTRODUCTION

Fetal heart rate (FHR) monitoring (the ‘non‐stress test’ or ‘cardiotocography’ (CTG)) is one of the most common obstetric investigations^1^. The patterns obtained are used to evaluate fetal brain health and overall wellbeing^2^, ^3^. Antepartum FHR monitoring is performed frequently to assess whether a fetus is at risk of an adverse outcome or death^4^, ^5^.

The reproducibility and reliability of human visual analysis of FHR traces are poor^6^. Expert clinical evaluation fails to identify accurately 35–92% of FHR patterns^7^, ^8^. Inter‐ and intraobserver agreement between experts is estimated to be as low as 29%, and false‐positive rates for identifying a fetus at risk of an adverse outcome are as high as 60%^9^, ^10^, ^11^, ^12^, ^13^, ^14^. Misinterpretation of FHR traces has been implicated directly in avoidable pregnancy intervention and increased incidence of adverse pregnancy outcomes (APOs)^15^, ^16^, ^17^, ^18^.

Numerical analysis of the FHR trace was used by Profs. Dawes and Redman to develop a set of 10 criteria for identifying fetal wellbeing, known as the Dawes–Redman criteria^19^, ^20^. If all criteria are fulfilled, the trace is classified as ‘criteria met’ and the fetus is deemed ‘healthy’. If these criteria have not been met after 60 min, the trace is categorized as ‘criteria not met’ and advice is given to the operator to seek expert opinion. The Dawes–Redman algorithm is ubiquitous in global antepartum FHR monitoring; it is used in over 120 hospitals in the UK and has been incorporated into local and national clinical guidelines^19^, ^21^, ^22^.

In the present study, we undertook the first evaluation of the Dawes–Redman algorithm for its intended purpose at term: identifying antepartum fetuses in a state of wellbeing. We utilized clinical data from the last 30 years of use of the algorithm at the John Radcliffe Hospital in Oxford, UK, to perform a retrospective case–control study. We evaluated antepartum FHR traces acquired at term in two cohorts: pregnancies with normal pregnancy outcomes (NPOs) and those with high‐risk APOs. The performance of the algorithm in identifying antepartum fetuses with good wellbeing was evaluated. We assessed the general performance of the algorithm between 0 h and 48 h prior to delivery and whether temporal proximity to delivery and distinct adverse outcomes affected these results.

## METHODS

Raw digital antepartum FHR traces obtained between 1 January 1991 and 1 February 2024 were extracted from the Oxford University Hospitals maternity database at the John Radcliffe Hospital (Oxford, UK). Traces were acquired from singleton pregnancies between 37 + 0 and 41 + 6 weeks' gestation, for which associated clinical outcome information was available for the mother and the newborn. Each trace had undergone Dawes–Redman analysis previously as part of standard clinical practice. Traces missing > 30% of their signal information or with incomplete Dawes–Redman analysis were removed. This study was approved by the Ethics Committee in the Joint Research Office, Research and Development Department, Oxford University Hospitals NHS Trust (approval number: 13/SC/0153).

The APOs considered in this study were acidemia, stillbirth, asphyxia, extended special care baby unit (SCBU) admission, hypoxic‐ischemic encephalopathy (HIE), low Apgar scores (1‐min Apgar score < 4; 5‐min Apgar score < 7) and neonatal resuscitation at delivery (including cardiac massage and tromethamine/bicarbonate administration). These outcomes were selected due to their significant association with neonatal health and the necessity for prompt identification to improve clinical outcomes (Table S1).

Antepartum FHR traces acquired within 48 h before delivery were selected for inclusion in the NPO and APO cohorts. As FHR monitoring is performed for varying clinical indications, it is not definitively reliable to assume that monitoring performed throughout a pregnancy pertains to a consistent clinical indication. Including traces that were collected at a time substantially prior to the development of an adverse outcome, without corroborating clinical evidence, would imply all traces were conducted under similar pathological conditions. Constraining the timeframe to within 48 h before delivery assists in mitigating this assumption that traces were obtained under similar pathological conditions. This approach aligns with clinical practice, in which FHR monitoring outcomes are used to inform immediate decision‐making.

Pregnancies were included in the NPO cohort if they met the following criteria: liveborn singleton neonate, gestational age at delivery between 37 + 0 and 41 + 6 weeks, maternal age at presentation between 18 and 50 years, birth weight ≥ 10^th^ centile, duration of labor < 24 h, 1‐min and 5‐min Apgar scores of ≥ 4 and ≥ 7, respectively, and umbilical venous and/or arterial pH within the normal range (arterial pH > 7.13 and base deficit < 10.0 mmol/L for neonates delivered via Cesarean section without labor; arterial pH > 7.05 and base deficit < 14.0 mmol/L for neonates who experienced labor). Additionally, a cerebroplacental ratio > 1.5 at 36 weeks' gestation was required for inclusion, if these data were available^23^.

Pregnancies were excluded from the NPO cohort for any of the following reasons: neonatal death within 3 months, emergency Cesarean section, breech presentation, need for neonatal resuscitation, SCBU admission following delivery or neonatal cooling, hypertensive disorders of pregnancy such as pre‐eclampsia, and clinically suspected infection or sepsis.

Pregnancies from the NPO cohort were matched with those from the APO cohort using one‐to‐one propensity score matching (PSM). Matching controlled for six factors: gestational age at FHR monitoring, fetal sex, maternal body mass index (BMI) at presentation, maternal age at delivery, parity and interval between FHR trace collection and delivery. Controlling for these variables ensured that each case in the NPO cohort had a directly comparable counterpart in the APO cohort, minimizing potential bias. See Appendix S1 for a description of each factor controlled for in PSM.

Propensity scores were estimated using logistic regression, chosen for its efficiency in handling binary outcomes and robustness in adjusting for confounders^24^. The outcome assignment was coded as 1 for the APO cohort and 0 for the NPO cohort. Interaction terms were included to examine if the effect of one covariate on the pregnancy outcome changes depending on another covariate. Propensity scores were matched using a Nearest Neighbors model, implemented with a Ball Tree algorithm to facilitate efficient searches in high‐dimensional spaces. A caliper of 0.05, reflecting 5% of the standard deviation of the logit of the propensity score, was applied to enhance matching specificity and quality. Matching quality was assessed using several metrics to ensure balanced cohorts. Standardized mean differences (SMDs) were used to measure covariate balance, with a SMD of less than 0.10 indicating an acceptable balance. Histograms, bar charts, the area under the receiver‐operating‐characteristics curve (AUC) and Brier scores were used to evaluate matching effectiveness^24^.

### Statistical analysis

Dawes–Redman algorithm results were considered ‘positive’ when the algorithm assigned the FHR trace as ‘criteria not met’ and ‘negative’ when designated as ‘criteria met’ (i.e. the fetus was in a state of wellbeing). A pregnancy was labeled as ‘positive’ if it aligned with the APO cohort and ‘negative’ otherwise. True positives refer to cases where both the antepartum FHR trace and the pregnancy status were classified as positive. False positives occurred when the FHR trace was deemed as ‘criteria not met’ despite the pregnancy belonging to the NPO cohort. True negatives occurred when the algorithm identified the trace as ‘criteria met’ in NPO pregnancies, while false negatives represent cases in which the pregnancy was associated with the APO cohort, yet the algorithm classified the trace as ‘criteria met’, i.e. negative.

Accuracy, sensitivity, specificity, positive predictive value (PPV) and negative predictive value (NPV) were calculated for each analysis (definitions and detailed descriptions are provided in Table S2). Accurate appraisal of the PPV and NPV is dependent on the underlying prevalence of disease within a given population, which was the APO cohort in the present study^25^. For clinical contextualization, we evaluated the PPV and NPV across the following theoretical risk strata: APO prevalence of 1% (very low risk), 10% (low risk), 20% (medium risk) and 30% (high risk). For subgroup analysis, we used a prevalence of 5%. This enabled adjustment of the PPV and NPV according to the specified prevalence, facilitating contextualization of the algorithm's performance in scenarios more aligned with obstetric practice.

Three analyses were conducted. We first evaluated the performance of the Dawes–Redman algorithm for all term antepartum FHR traces acquired between 0 h and 48 h prior to delivery. We then evaluated whether temporal proximity to delivery affected performance. Data were partitioned into two time windows: between 0 h and 23 h 59 mins prior to delivery (0–24 h) and between 24 h and 48 h prior to delivery. We then compared the performance of the algorithm between the two time windows. Finally, we investigated if performance differed meaningfully by APO. We calculated the performance metrics for each individual outcome belonging to the APO cohort separately for all traces between 0 and 48 h.

Conditional logistic regression was used to assess the association between the Dawes–Redman algorithm output and pregnancy outcomes, accounting for the matched nature of the data. The model was fitted using maximum likelihood estimation, optimizing the log‐likelihood function to obtain parameter estimates that best explain the association between the algorithm's output and pregnancy outcomes.

Categorical variables are presented as *n* (%) and continuous variables are given as median (interquartile range (IQR)). Performance metrics are expressed in percentages. Categorical variables were compared using the chi‐square test and continuous variables were compared using the Mann–Whitney *U*‐test, with a threshold of *P* < 0.05 used to indicate significance. Sensitivity, specificity, PPV and NPV were calculated using standard methods^26^. Confidence intervals for PPV and NPV were calculated using the method described by Mercaldo *et al*.^27^. Analysis was performed using Python version 3.9.17 (www.python.org), with the Pandas (version 1.5.3), NumPy (version 1.23.5), Matplotlib (version 3.7.1) and SciPy (version 1.10.1) packages.

## RESULTS

In this study, 1658 FHR traces from pregnancies with reported APOs were matched successfully with 1658 traces from pregnancies with reported NPO, resulting in 3316 FHR records. PSM successfully created well‐balanced cohorts with an AUC of 0.67 (95% CI, 0.64–0.69), overall SMD of < 0.10 and a Brier score of < 0.01 (Figure S1). The median gestational age at delivery was 39 (IQR, 38–40) weeks for both the APO and NPO cohorts, with a SMD of 0.00. The fetal sex ratio was 56.6% male for both cohorts (SMD = 0.00). The median maternal BMI at presentation was 25.9 (IQR, 22.6–30.1) kg/m^2^ for the APO cohort and 25.7 (IQR, 22.7–30.1) kg/m^2^ for the NPO cohort (SMD = 0.08). The median maternal age at delivery was 31.0 (IQR, 27.0–35.0) years in both the APO and NPO cohorts (SMD = 0.00). The median gravidity was 1 (IQR, 0–1) for APO and 1 (IQR, 0–2) for NPO, and the median parity was 0 (IQR, 0–1) for both cohorts (SMD = 0.00). With respect to type of labor, 54.6% of the APO cohort underwent induced labor compared to 46.8% in the NPO cohort, with spontaneous labor accounting for 27.0% of pregnancies in the APO and 36.8% of pregnancies in the NPO cohort. Delivery methods also differed, with spontaneous delivery more common in the NPO cohort (57.4%) compared with the APO cohort (31.9%).

Birth weights were similar between cohorts: the median male birth weight was 3405.0 (IQR, 2960.2–3830.0) g for the APO cohort and 3440.0 (IQR, 3121.0–3739.0) g for the NPO cohort, while the median female birth weight was 3268.5 (IQR, 2940.0–3647.0) g for the APO cohort and 3270.0 (IQR, 3004.5–3598.5) g for the NPO cohort. The 1‐min Apgar scores were notably lower in the APO cohort, with a median of 3.0 (IQR, 2.0–8.0) compared to 10.0 (IQR, 9.0–10.0) in the NPO cohort. At 5 min, the median Apgar score in the APO cohort improved to 9.0 (IQR, 6.0–10.0) compared to 10.0 (IQR, 10.0–10.0) in the NPO cohort. The median umbilical artery pH was 7.19 (IQR, 7.08–7.27) in the APO cohort and 7.24 (IQR, 7.18–7.29) in the NPO cohort. Median umbilical vein pH levels were 7.29 (IQR, 7.21–7.35) in the APO cohort and 7.33 (IQR, 7.28–7.36) in the NPO cohort. Arterial base excess was higher in the APO cohort, with a median of 6.90 (IQR, 4.00–10.90) mmol/L compared to 5.70 (IQR, 3.70–8.10) mmol/L in the NPO cohort, and median venous base excess was also higher in the APO cohort at 5.10 (IQR, 2.92–8.10) mmol/L compared to 4.20 (IQR, 2.60–6.20) mmol/L in the NPO cohort. Median lactate levels were elevated slightly in the APO cohort at 4.5 (IQR, 3.1–6.4) mmol/L compared with 4.3 (IQR, 3.2–5.5) mmol/L in the NPO cohort. Maternal and neonatal characteristics are presented in Tables S3 and S4, respectively. Table S5 describes the frequency of each adverse outcome.

### Performance evaluation of Dawes–Redman algorithm

The conditional multinomial logistic regression model examined the association between the Dawes–Redman algorithm output (‘criteria met’ or ‘criteria not met’) and pregnancy outcomes (NPO or APO). The results demonstrated a statistically significant relationship between the Dawes–Redman algorithm output and APOs. The coefficient for the algorithm's result was 0.80 (95% CI, 0.58–1.02; *P* < 0.001), indicating that failure to meet the criteria was associated with an increased risk of APO. Exponentiating this coefficient gave an odds ratio of approximately 2.23, suggesting that not meeting the Dawes–Redman criteria is associated with a 123% increase in the odds of APO.

Of the 1658 FHR traces in the NPO cohort, 1503 (90.7%) were identified correctly as ‘criteria met’ indicating fetal wellbeing, while 155 (9.3%) were identified incorrectly as ‘criteria not met’. Of the 1658 FHR traces in the APO cohort, 301 (18.2%) were identified correctly as ‘criteria not met’, while 1357 (81.8%) were identified incorrectly as ‘criteria met’ (Table 1). This resulted in a specificity of 90.7% (95% CI, 89.2–92.0%), indicating a high true‐negative rate, while the sensitivity was 18.2% (95% CI, 16.3–20.0%), indicating a low true‐positive rate. The overall accuracy of the algorithm was 54.4% (95% CI, 52.0–56.8%) (Table 2).

**Table 1:** Confusion matrix for Dawes–Redman analysis of fetal heart rate (FHR) traces obtained at term in pregnancies with normal and those with adverse pregnancy outcome

	Pregnancy outcome		
Dawes–Redman result	Adverse	Normal	Total
Criteria not met	301	155	456
Criteria met	1357	1503	2860
Total	1658	1658	3316

Data are given as *n*.

Datasets were matched for gestational age at FHR monitoring, fetal sex, maternal age at delivery, maternal body mass index at presentation, parity and time interval between FHR monitoring and delivery, resulting in a balanced dataset of 1658 traces in each cohort.

‘Criteria met’ indicates good fetal wellbeing.

**Table 2:** Performance of the Dawes–Redman algorithm in interpreting fetal heart rate traces from 3316 pregnancies with subsequent normal (NPO) or adverse (APO) pregnancy outcome at delivery

Performance metric	% (95% CI)
Accuracy	54.4 (52.0–56.8)
Sensitivity	18.2 (16.3–20.0)
Specificity	90.7 (89.2–92.0)
Theoretical risk group for APO*	
Very low risk	
PPV	1.9 (1.6–2.4)
NPV	99.1 (98.9–99.3)
Low risk	
PPV	17.7 (14.9–21.0)
NPV	90.9 (89.0–92.5)
Medium risk	
PPV	32.7 (28.3–37.4)
NPV	81.6 (78.3–84.5)
High risk	
PPV	45.4 (40.4–50.6)
NPV	72.1 (67.7–76.1)

* Four theoretical risk groups were evaluated to demonstrate how the prevalence of APO affects performance: very low risk (1% prevalence of APO, 99% prevalence of NPO), low risk (10% prevalence of APO, 90% prevalence of NPO), medium risk (20% prevalence of APO, 80% prevalence of NPO) and high risk (30% prevalence of APO, 70% prevalence of NPO).

NPV, negative predictive value; PPV, positive predictive value.

As the prevalence of APOs increased, the PPV and NPV of the Dawes–Redman algorithm changed substantially. At an APO prevalence of 1%, the PPV was 1.9% (95% CI, 1.6–2.4%) and the NPV was 99.1% (95% CI, 98.9–99.3%), indicating a high probability of identifying NPOs correctly. With an APO prevalence of 10%, the PPV increased to 17.7% (95% CI, 14.9–21.0%) while the NPV decreased to 90.9% (95% CI, 89.0–92.5%). At a 20% prevalence of APO, the PPV rose to 32.7% (95% CI, 28.3–37.4%) and the NPV declined to 81.6% (95% CI, 78.3–84.5%). At an APO prevalence of 30%, the PPV was maximal at 45.4% (95% CI, 40.4–50.6%) while the NPV was 72.1% (95% CI, 67.7–76.1%) (Table 2).

### Temporal proximity of FHR assessment to delivery

The difference in the Dawes–Redman algorithm's discriminatory power when comparing FHR traces acquired at 0–24 h and at 24–48 h prior to delivery was evaluated (Table 3). The algorithm demonstrated significantly higher accuracy within the 0–24‐h window (56.9% (95% CI, 53.6–60.3%)) compared with the 24–48‐h window (51.8% (95% CI, 48.4–55.2%)) (*P* = 0.008). Specificity remained high for both intervals (90.8% (95% CI, 88.8–92.7%) for 0–24 h and 90.3% (95% CI, 88.2–92.3%) for 24–48 h) without significant variation (*P* = 0.898). However, sensitivity decreased from 23.0% (95% CI, 20.2–25.9%) in the 0–24‐h interval to 13.2% (95% CI, 10.9–15.6%) in the 24–48‐h interval (*P* = 0.001). The PPV in the 0–24‐h interval was 11.7% (95% CI, 9.0–14.9%) and the NPV was 95.7% (95% CI, 94.4–96.8%) when the APO prevalence was set at 5%. For FHR traces acquired between 24 h and 48 h prior to delivery, the PPV was lower at 6.7% (95% CI, 5.0–9.0%) and the NPV was 95.2% (95% CI, 93.6–96.4%).

**Table 3:** Comparison of performance of the Dawes–Redman algorithm in identifying pregnancies with subsequent normal or adverse pregnancy outcome at delivery* when fetal heart rate assessment was performed 0–24 h *vs* 24–48 h before delivery

	Time before delivery		
Performance metric	0–24 h	24–48 h	*P*
Accuracy	56.9 (53.6–60.3)	51.8 (48.4–55.2)	0.008
Sensitivity	23.0 (20.2–25.9)	13.2 (10.9–15.6)	0.001
Specificity	90.8 (88.8–92.7)	90.3 (88.2–92.3)	0.898
PPV	11.7 (9.0–14.9)	6.7 (5.0–9.0)	0.006
NPV	95.7 (94.4–96.8)	95.2 (93.6–96.4)	0.079

Data are given as % (95% CI).

* A theoretical base rate of 5% adverse pregnancy outcome and 95% normal pregnancy outcome prevalence was applied.

NPV, negative predictive value; PPV, positive predictive value.

### Performance of Dawes–Redman algorithm by adverse outcome

The ability of the Dawes–Redman algorithm to identify fetal wellbeing varied across different adverse outcomes (Table S6). Accuracy ranged from 53.8% (95% CI, 47.1–60.6%) for acidemia to 59.7% (95% CI, 46.8–71.0%) for neonatal resuscitation. Sensitivity was generally low, from 16.8% (95% CI, 12.0–22.1%) for acidemia to 25.8% (95% CI, 16.1–37.1%) for neonatal resuscitation. Specificity was higher, ranging from 87.7% (95% CI, 84.6–90.5%) for extended SCBU admission to 94.7% (95% CI, 87.7–100.0%) for HIE. The PPV varied across adverse outcomes, from 8.2% (95% CI, 6.0–11.2%) for extended SCBU admission to 18.6% (95% CI, 5.8–46.0%) for HIE. Conversely, the NPV was high across all outcomes, ranging from 95.4% (95% CI, 92.0–97.4%) for acidemia to 96.0% (95% CI, 88.2–98.7%) for neonatal resuscitation.

## DISCUSSION

We detail a comprehensive performance evaluation of the Dawes–Redman algorithm in pregnancies at term and describe the limitations of the algorithm. The Dawes–Redman algorithm is currently integrated into electronic FHR monitoring systems by principal manufacturers on a global scale^28^, ^29^, ^30^. In the UK, this algorithm is utilized in upwards of 120 healthcare institutions and is internationally distributed to over 110 nations. This system is employed routinely in clinical decision‐making. However, there has been scant analysis of its performance, despite its prominence in obstetric care and national guidelines^21^.

The results described in the present study provide crucial insights into obstetric clinical practice. While the accuracy of the Dawes–Redman algorithm (namely, its ability to correctly discern between FHR traces from pregnancies with NPO and those with APO) is relatively low, its specificity (i.e. ability to rule out APOs) is high, exceeding 90% on average. When the Dawes–Redman criteria are met, it is a reliable indicator of fetal wellbeing and that high‐risk pathology (such as the adverse outcomes defined in this study) is not present. This was also demonstrated across the array of adverse outcomes and with an increasing interval between FHR monitoring and delivery. This was the original intended purpose of the Dawes–Redman algorithm. The sensitivity of the algorithm (i.e. ability to identify correctly pregnancies with an adverse outcome) is low. Failing to meet the Dawes–Redman criteria is associated with an increase in the odds of APO. This underscores the importance of interpreting ‘criteria not met’ results with caution and ensuring further assessment of the fetus by other modalities. The Dawes–Redman algorithm was not originally developed, and therefore not intended, to identify pregnancies at risk of APO. Thus, a high degree of caution should be maintained when interpreting any FHR trace failing to meet the Dawes–Redman criteria. Importantly, a Dawes–Redman antepartum CTG analysis that does meet the criteria could occur in a pregnancy that subsequently develops an APO at any time after the FHR assessment, for example during labor. This possibly explains the false‐negative results observed in this study. For this reason, current advice is that these results are valid only for the period in which they were performed. The Dawes–Redman criteria are also strict. Failure to meet the criteria can occur due to approximately 60 unique reasons, ranging from low risk (recording artifacts identified at the end of a FHR trace) to immediate high risk (presence of a high‐frequency sinusoidal rhythm)^20^.

The underlying prevalence of APO in the obstetric population is crucial and varies across clinical environments and practices, dependent on factors such as obstetric history, clinical presentation, examination and even the clinic that the pregnant woman is attending. Both PPVs and NPVs are dependent on the prevalence of the outcome of interest. The NPV in the very‐low‐risk population (99% prevalence of NPO) was greater than 99% and the PPV consistently exceeded the base rate for APOs. We have also shown that predictive performance declines when the population prevalence of fetal wellbeing decreases. In a high‐risk cohort (30% prevalence of APO and 70% prevalence of NPO), the NPV decreased to 71.5%. In this scenario, the utility of the Dawes–Redman system, while high, is relatively more limited. It is important that users of this algorithm are aware of this. We observed that NPVs were near the base rate for NPO across these strata. It is important to remember that approximating a base rate does not inherently indicate poor predictive value, especially when contrasting performance with human visual interpretation of FHR traces, for which the rates of false positives and false negatives are known to be high. For example, the Dawes–Redman algorithm confirming that a FHR trace indicates a > 99% probability of fetal wellbeing with NPO is still of substantial clinical value. The prevalence rates used in this study are theoretical. While it is neither reasonable nor pragmatic for the healthcare provider to know exact prevalences in routine practice, this study demonstrates why appreciating the effect of prevalence is paramount. We have therefore developed the following conclusions and recommendations. (1) The Dawes–Redman algorithm performs well in its intended purpose: identifying a fetus in a state of wellbeing, i.e. not at immediate risk of an adverse outcome. (2) The diagnostic performance of any clinical investigation, specifically PPV and NPV, is dependent on the underlying probability (i.e. prevalence) of the outcome of interest. Modeling the effect of prevalence on the performance of the algorithm confirms this is just as true for the Dawes–Redman algorithm. The practitioner should consider the pretest probability of normality on a case‐by‐case basis when interpreting the results of this system. (3) Failure of pregnancies to meet the Dawes–Redman criteria is of limited clinical utility in the context of predicting APO. The 10 Dawes–Redman criteria are strict. Failure to meet these criteria is a non‐specific indicator of marginally increased risk. Expert clinical opinion always supersedes the Dawes–Redman algorithm. Any doubt or uncertainty surrounding the wellbeing of a fetus should always be an indication to the practitioner to seek expert obstetric opinion, regardless of the result from the Dawes–Redman algorithm. (4) There is a pressing need for the development of FHR analysis algorithms aimed at identifying at‐risk or high‐risk pregnancies.

Previous studies have investigated the significance of failing to meet the Dawes–Redman criteria in high‐risk pregnancies and how such cases should be interpreted. Bhide *et al*.^31^ conducted a retrospective observational study, finding that approximately 6–7% of pregnancies failed to meet the Dawes–Redman criteria, with these cases exhibiting an 8‐ to 9‐fold increased risk of stillbirth compared to those meeting the criteria. Stampalija *et al*.^32^ focused on the application of computerized CTG incorporating the Dawes–Redman criteria, particularly addressing how unmet criteria should be managed. Both studies have limitations in their approaches. For example, Bhide *et al*.^31^ did not adjust for confounding variables, introducing potential biases, while Stampalija *et al*.^32^ relied on narrative synthesis and anecdotal evidence, lacking robust prospective datasets.

Our study addressed these gaps by employing PSM to create balanced cohorts, enabling a more rigorous evaluation of predictive performance metrics. While Stampalija *et al*. raised concerns about overmedicalization due to false‐positive results^32^, our findings highlight the importance of confounder adjustment to interpret accurately the predictive significance of criteria failure. Despite these differences, these studies agree on the high specificity of the Dawes–Redman algorithm in identifying healthy pregnancies and the necessity of careful clinical reassessment when criteria are not met. Both analyses^31^, ^32^ conclude that computerized CTG should not be used in isolation, particularly in high‐risk pregnancies, and highlight the need for further evidence‐based approaches to optimize its clinical application. These complementary insights advance the understanding of the role of computerized CTG in antenatal care.

This was a rigorous, large‐scale study; however, there are important limitations to consider. While the data were retrospective, acquired over 30 years of obstetric care using a variety of FHR monitoring devices from different manufacturers, a multicenter, ideally international, study would further bolster these results. We also note that the longevity of a Dawes–Redman ‘criteria met’ result is not yet known. Therefore, the results of this study should not be extrapolated beyond the 48‐h window we have examined here. This study utilized retrospective case–control data. While a dedicated team of midwives and clinicians validated and entered these routine clinical data into a dedicated study database on a weekly basis, the gold standard would be a prospective multicenter study. Given the relative paucity of certain adverse obstetric outcomes (e.g. stillbirth occurs in 3.9 births per 1000 total births in the UK), we estimate the time to accumulate the required sample size needed for such an undertaking would be approximately 8–10 years. We have already begun such an undertaking at international obstetric hospitals.

## Supporting information


**Table S1** Definitions and justifications of adverse outcomes utilized for inclusion of cases in adverse pregnancy outcome cohort
**Table S2** Definitions of performance metrics used for evaluation of the Dawes–Redman algorithm
**Table S3** Maternal and labor characteristics, according to normal pregnancy outcome or adverse pregnancy outcome
**Table S4** Neonatal characteristics, according to normal pregnancy outcome or adverse pregnancy outcome
**Table S5** Frequency of adverse pregnancy outcomes and associated number of fetal heart rate traces recorded
**Table S6** Performance of the Dawes–Redman algorithm across 10 adverse pregnancy outcomes in fetal heart rate traces acquired within 48 h before delivery
**Appendix S1** Description of six factors that were controlled for propensity score matching
**Figure S1** Histograms and bar charts for each variable used in propensity score matching to develop balanced cohorts of adverse pregnancy outcome (APO) and normal pregnancy outcome (NPO).

## Data Availability

The authors acknowledge the importance of data transparency and the potential value of data sharing in advancing scientific research. However, due to the identifiable and sensitive nature of the data used in this study, which includes detailed fetal heart rate traces potentially linked to individual patient outcomes, we are unable to make the dataset publicly available. The data contains protected health information and is subject to strict confidentiality constraints to safeguard the privacy of individuals. Consequently, the ethical and legal restrictions prevent the sharing of the dataset.
