# Supplementary material for: Performance evaluation of computerized antepartum fetal heart rate monitoring: Dawes–Redman algorithm at term
Source: Ultrasound Obstet Gynecol. 2025 Feb 2;65(2):191–7. doi: 10.1002/uog.29167 (PMC11788457; doi:10.1002/uog.29167)
Supplement: Supplementary file 1 — Table S1 Definitions and justifications of adverse outcomes utilized for inclusion of cases in adverse pregnancy outcome cohort Table S2 Definitions of performance metrics used for evaluation of the Dawes–Redman algorithm Table S3 Maternal and labor characteristics, according to normal pregnancy outcome or adverse pregnancy outcome Table S4 Neonatal characteristics, according to normal pregnancy outcome or adverse pregnancy outcome Table S5 Frequency of adverse pregnancy outcomes and associated number of fetal heart rate traces recorded Table S6 Performance of the Dawes–Redman algorithm across 10 adverse pregnancy outcomes in fetal heart rate traces acquired within 48 h before delivery Appendix S1 Description of six factors that were controlled for propensity score matching Figure S1 Histograms and bar charts for each variable used in propensity score matching to develop balanced cohorts of adverse pregnancy outcome (APO) and normal pregnancy outcome (NPO). [file UOG-65-191-s001.docx]

| Adverse Outcome | Definition | Justification |
| --- | --- | --- |
| Acidaemia | Two sets of acidaemia values are used: Babies delivered by CS without labour: arterial pH <7.13 AND arterial BD >10.0; Babies who experienced labour (regardless of delivery method): arterial pH <7.05 and arterial BD >14.0. | Fetal acidaemia indicates a significant imbalance of pH levels in the fetus/neonate’s blood, typically signifying a lack of oxygen. This condition can result in immediate threats to the infant's survival and long-term neurological damage if not promptly identified and treated. |
| Birth Asphyxia | Low Apgar score(s) + Acidaemia  *Low Apgar score(s) are defined below* | Birth asphyxia occurs when there is an insufficient supply of oxygen to the baby before, during, or shortly after birth, leading to hypoxia and acidosis. This condition can cause organ failure and severe neurological issues, requiring immediate medical intervention to prevent lifelong disability or death. |
| Extended SCBU admission | Neonates born at or after 37^+0^ gestational weeks who were admitted for at least 7 days to SCBU or NICU. | A prolonged stay in a special care unit indicates significant health issues requiring close medical attention, such as respiratory distress syndrome or severe infections. The need for extended SCBU care reflects the severity of the infant's condition, impacting their development and long-term health. |
| Hypoxic ischaemic encephalopathy (HIE) | Diagnosed by treating clinical team (neonatal/paediatrics). | HIE is severe brain damage resulting from a lack of oxygen and blood flow to the brain, leading to immediate consequences like seizures and death, and long-term debilitating effects on developmental and cognitive abilities. Early diagnosis and intervention are crucial to mitigate the effects of HIE and improve the infant’s prognosis. |
| Low Apgar score(s) | Apgar score <4 at 1 minute; Apgar score <7 at 5 minutes. | The Apgar score assesses a newborn's heart rate, reflexes, muscle tone, skin colour, and respiration. A low Apgar score requires immediate medical intervention and potentially resuscitation, serving as a proxy for neonatal well-being and future health. |
| Neonatal Resuscitation | Either cardiac massage, sodium bicarbonate or and tris‐hydroxymethyl‐aminomethane (THAM) used following delivery of the fetus. | Neonatal resuscitation is an emergency procedure used to restore normal heart rate and breathing in a newborn experiencing life-threatening conditions such as severe acidosis, respiratory distress, or cardiac arrest. Immediate resuscitation is critical to prevent organ damage, neurological injury, or death. Interventions such as cardiac massage, sodium bicarbonate to counteract metabolic acidosis, and THAM to buffer blood pH are essential in stabilizing the infant. These actions are vital in managing neonates who fail to establish spontaneous respiration or normal circulation, ensuring better outcomes in the critical post-delivery period. |
| Stillbirth | Antepartum or intrapartum stillbirth, as diagnosed by treating clinical team. | Stillbirth, occurring before or during labour, or death within 7 days of delivery is a severe outcome with profound impact. Identifying risk factors early can lead to interventions that may save lives, making it a crucial high-risk condition for healthcare providers to monitor and manage through fetal monitoring and timely intervention. |
| Supplementary Table 1: Definitions and justifications of adverse outcomes utilized for inclusion of cases in adverse pregnancy outcome cohort. Each outcome is selected based on its significant impact on or association with neonatal health and the necessity for prompt identification and intervention to improve clinical outcomes. Detailed justifications are provided to underline the importance of monitoring and managing these conditions to ensure better perinatal outcomes. | | |

| Metric | Description |
| --- | --- |
| Accuracy | The proportion of total classifications (both APO and NPO) that are correct. Evaluates how accurately the algorithm classifies FHR traces and aligns them with the corresponding pregnancy outcome. |
| Sensitivity | The ability of the algorithm to correctly identify 'APO' cases, i.e., instances where both the FHR trace is classified as ‘criteria not met’ and the pregnancy outcome is adverse. |
| Specificity | The algorithm's capability to correctly identify 'NPO' cases, where both the FHR trace is classified as 'criteria met' and the pregnancy belongs to the normal outcome cohort. |
| Positive Predictive Value (PPV) | The probability that an 'APO' result (FHR trace classified as 'criteria not met') corresponds to an adverse pregnancy outcome. |
| Negative Predictive Value (NPV) | The probability that an 'NPO' result (FHR trace classified as 'criteria met') corresponds to a normal pregnancy outcome. |
| Supplementary Table 2: Definitions of performance metrics used for evaluation of Dawes–Redman algorithm | |

|  | NPO | APO |  |
| --- | --- | --- | --- |
| Maternal age at delivery (years) | 31.0 (27.0-35.0) | 31.0 (27.0-35.0) |  |
| Gravidity | 1 (0-2) | 1 (0-1) |  |
| Parity | 0 (0–1) | 0 (0–1) |  |
| Maternal BMI at booking (kg/m^2^) | 25.7 (22.7-30.1) | 25.9 (22.6-30.1) |  |
| Labour Type (N, %) | | |  |
| Spontaneous | 610 (36.8%) | 448 (27.0%) |  |
| Induced | 776 (46.8%) | 906 (54.6%) |  |
| No labour | 269 (16.2%) | 299 (18.0%) |  |
| Delivery Method (N, %) | | | |
| Spontaneous delivery | | 952 (57.4%) | 529 (31.9%) |
| Ventouse extraction | | 133 (8.0%) | 80 (4.8%) |
| Forceps delivery | | 303 (18.3%) | 338 (20.4%) |
| Emergency caesarean section | | 0 (0.0%) | 393 (23.7%) |
| Elective caesarean section | | 270 (16.3%) | 299 (18.0%) |
| Breech | | 0 (0.0%) | 19 (1.1%) |
| Supplementary Table 3: Maternal and labor characteristics, according to normal pregnancy outcome cohort or adverse pregnancy outcome cohort. For each antepartum fetal monitoring record from an adverse pregnancy outcome (APO), a corresponding record from a normal pregnancy outcome (NPO) was identified using propensity score matching, balanced for gestational age at recording, fetal sex, maternal BMI at booking, maternal age at delivery, parity and time the FHR trace was recorded prior to delivery. In total, 3,980 FHR monitoring records were identified. | | |  |

|  | NPO | APO |  |
| --- | --- | --- | --- |
| Gestational age at delivery | 39.0 (38.0-40.0) | 39.0 (38.0-40.0) |  |
| Male | 939 (56.6%) | 938 (56.6%) |  |
| Female | 719 (43.4%) | 720 (43.4%) |  |
| Male birthweight (grams) | 3,440.0 (3121.0-3739.0) | 3,405.0 (2960.2-3830.0) |  |
| Female birthweight (grams) | 3,270.0 (3004.5-3598.5) | 3,268.5 (2940.0-3647.0) |  |
| Apgar at 1 minute | 10.0 (9.0-10.0) | 3.0 (2.0-8.0) |  |
| Apgar at 5 minutes | 10.0 (10.0-10.0) | 9.0 (6.0-10.0) |  |
| Apgar at 10 minutes | 10.0 (10.0-10.0) | 10.0 (9.0-10.0) |  |
| Umbilical Artery pH | | 7.24 (7.18-7.29) | 7.19 (7.08-7.27) |
| Umbilical Vein pH | | 7.33 (7.28-7.36) | 7.29 (7.21-7.35) |
| Arterial Base excess | | 5.70 (3.70-8.10) | 6.90 (4.00-10.90) |
| Venous Base excess | | 4.20 (2.60-6.20) | 5.10 (2.92-8.10) |
| Lactate (mmol/L) | | 4.3 (3.2-5.5) | 4.5 (3.1-6.4) |
| Supplementary Table 4: Neonatal characteristics, according to normal pregnancy outcome cohort or adverse pregnancy outcome cohort. NPO = normal pregnancy outcome; APO = adverse pregnancy outcome, comprising at least one of the adverse outcomes detailed in Supplementary Table 1. | | |  |

| Adverse Pregnancy Outcome | FHR traces |
| --- | --- |
| Acidaemia | 208 |
| Asphyxia | 59 |
| Extended SCBU admission | 495 |
| Hypoxic Ischaemic Encephalopathy | 57 |
| Low Apgar score | 1,077 |
| Neonatal Resuscitation | 62 |
| Stillbirth | 40 |
| Supplementary Table 5: Frequency of adverse pregnancy outcomes and associated number of fetal heart rate traces recorded. Outcome definitions are provided in Supplementary Table 1. Values sum to >1,658 due to pregnancies with multiple adverse outcomes. | |

| Metric | Acidaemia | Asphyxia | Extended SCBU admission | Hypoxic ischaemic encephalopathy | Low Apgar score | Neonatal Resuscitation | Stillbirth and Early Neonatal Death |
| --- | --- | --- | --- | --- | --- | --- | --- |
| Accuracy | 53.8 (47.1–60.6) | 55.1 (42.4–67.8) | 54.3 (49.9–58.8) | 58.8 (45.6–71.9) | 55.3 (52.4–58.3) | 59.7 (46.8–71.0) | 56.2 (40.0–72.5) |
| Sensitivity | 16.8 (12.0–22.1) | 18.6 (8.5–28.8) | 21.0 (17.6–24.6) | 22.8 (12.3–33.3) | 18.9 (16.6–21.3) | 25.8 (16.1–37.1) | 20.0 (7.5–32.5) |
| Specificity | 90.9 (87.0–94.7) | 91.5 (83.1–98.3) | 87.7 (84.6–90.5) | 94.7 (87.7–100.0) | 91.7 (90.1–93.3) | 93.5 (87.1–98.4) | 92.5 (82.5–100.0) |
| PPV | 8.8 (5.1–15.0) | 10.4 (3.6–26.3) | 8.2 (6.0–11.2) | 18.6 (5.8–46.0) | 10.8 (8.5–13.6) | 17.4 (6.2–40.2) | 12.3 (3.3–36.5) |
| NPV | 95.4 (92.0–97.4) | 95.5 (87.4–98.5) | 95.5 (93.7–96.7) | 95.9 (86.2–98.9) | 95.6 (94.3–96.6) | 96.0 (88.2–98.7) | 95.6 (84.3–98.9) |
| Supplementary Table 6: Performance of Dawes–Redman algorithm across 10 adverse pregnancy outcomes in fetal heart rate traces acquired within 48 h before delivery | | | | | | | |

| 1. **Gestational Age at FHR Monitoring:** Gestational age directly influences FHR characteristics, with variations attributed to fetal developmental stages.^29-31^ 2. **Fetal Sex:** Male and female fetuses exhibit different physiological responses to intrauterine stress, leading to variations in FHR dynamics. Male fetuses tend to have more pronounced linear heart rate dynamics and less complex patterns compared to female fetuses, which exhibit higher values of nonlinear indices, reflecting more active cardiovascular regulation.^32,33^ 3. **Maternal BMI at Booking:** Overweight and obese women have a higher risk for gestational diabetes and hypertensive disorders, conditions that can lead to altered FHR variability and reactivity. Additionally, increased maternal adiposity can impact placental function, leading to chronic hypoxia and oxidative stress, which manifest as changes in baseline FHR and reduced variability. ^34-37^ 4. **Maternal Age at Delivery:** Maternal age impacts fetal development and pregnancy outcomes, with advanced maternal age associated with higher risks of chromosomal anomalies, gestational diabetes, preeclampsia, and placental problems.^38,39^ 5. **Parity:** Parity influences uterine vascular adaptation and placental blood flow, which in turn affect FHR characteristics. Multiparous women often experience different uterine and placental adaptations compared to nulliparous women, potentially leading to variations in FHR patterns. Parity is also associated with differences in labour progression and uterine contractions, which can further influence FHR during monitoring. ^40-44^ 6. **Time Prior to Delivery:** The time interval between the antepartum CTG and delivery can encompass significant changes in fetal condition, affecting the algorithm's predictive accuracy. CTGs taken closer to delivery more accurately reflect the immediate fetal status, while those taken earlier may not, potentially leading to misclassification. Thus, accounting for this time variable is essential to ensure a reliable and clinically relevant evaluation of the algorithm's diagnostic performance. |
| --- |
| Appendix S1: Description of six factors that were controlled for propensity score matching. |

| 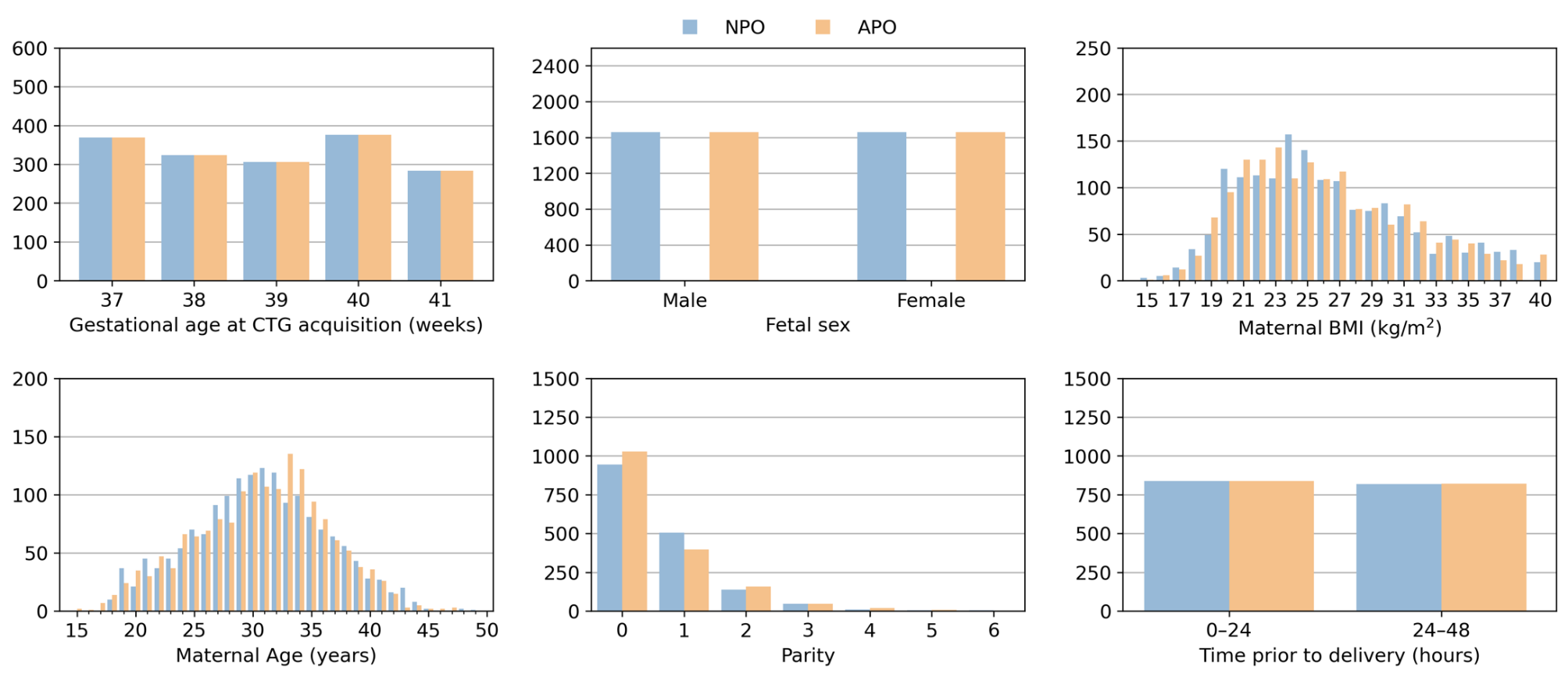 |
| --- |

| Supplementary Figure 1: Histograms and bar charts for each variable used in propensity score matching to develop balanced cohorts of adverse pregnancy outcome (APO) and normal pregnancy outcome (NPO). |
| --- |
